# *In Vitro* Androgen Bioassays as a Detection Method for Designer Androgens

**DOI:** 10.3390/s130202148

**Published:** 2013-02-06

**Authors:** Elliot R. Cooper, Kristine C. Y. McGrath, Alison K. Heather

**Affiliations:** 1 School of Medical and Molecular Biosciences, University of Technology, Sydney, Ultimo 2007, New South Wales, Australia; E-Mails: Elliot.R.Cooper@uts.edu.au (E.R.C.); Kristine.McGrath@uts.edu.au (K.C.Y.M.); 2 Heart Research Institute, Newtown 2042, Sydney, New South Wales, Australia

**Keywords:** androgens, bioassay, designer, doping, mammalian, nutraceutical, steroid, supplement, testosterone, yeast

## Abstract

Androgens are the class of sex steroids responsible for male sexual characteristics, including increased muscle mass and decreased fat mass. Illicit use of androgen doping can be an attractive option for those looking to enhance sporting performance and/or physical appearance. The use of *in vitro* bioassays to detect androgens, especially designer or proandrogens, is becoming increasingly important in combating androgen doping associated with nutritional supplements. The nutritional sports supplement market has grown rapidly throughout the past decade. Many of these supplements contain androgens, designer androgens or proandrogens. Many designer or proandrogens cannot be detected by the standard highly-sensitive screening methods such as gas chromatography-mass spectrometry because their chemical structure is unknown. However, *in vitro* androgen bioassays can detect designer and proandrogens as these assays are not reliant on knowing the chemical structure but instead are based on androgen receptor activation. For these reasons, it may be advantageous to use routine androgen bioassay screening of nutraceutical samples to help curb the increasing problem of androgen doping.

## 1. Introduction

Ever since the ancient Olympic Games, athletes have long sought the ability to enhance their performance in sports and continue to do so in the modern era of elite competition. Over the past few decades, athletes have attempted to enhance their performance with the use of exogenous hormones including androgens, erythropoietin and growth hormone. Androgens are the most effective form of sports doping and are the most common type of performance enhancing substances detected in screening tests. It was not until the 1972 Munich Olympic Games that the International Olympic Committee (IOC) introduced screening tests for exogenous androgens [1]. Since then, the World Anti-Doping Agency (WADA) enforces the banning of androgens through urine screening to detect even trace amounts of an extensive list of banned and prohibited substances which athletes are screened for prior, and during, competition. This list relies on screening urine samples with highly specific and sensitive techniques such as gas chromatography-mass spectrometry (GC-MS).

For some athletes, the prospect of success outweighs the health and unethical concerns of sports doping. In an attempt to bypass screening methods, designer androgens have been created that have different chemical structures to known androgens and, therefore, cannot be easily detected by GC-MS. However, because designer androgens have biological activity they activate the androgen receptor (AR), and therefore can be detected by androgen bioassays. Therefore, androgen bioassays may prove to be a suitable tool for routine screening of nutraceutical or biological samples suspected to contain an androgen.

## 2. Steroid Hormones

Steroid hormones include the sex hormones, glucocorticoids, and mineralocorticoids. Within the family of sex hormones are the androgens, estrogens, and progestogens. All of the steroid hormones bind to their own specific receptor, which may be cytosolic or nuclear, to induce changes within a cell. All natural steroid hormones are synthesized from cholesterol in the adrenal glands and/or gonads. Some steroid hormones are further metabolized in the liver, peripheral and/or target tissues. As their precursor is cholesterol, they are hydrophobic in nature which allows them to pass easily through cell membranes. Once synthesized, the steroid hormones are carried in the blood stream bound to carrier proteins such as albumin, steroid hormone-binding globulin (SHBG) or corticosteroid-binding globulin to target tissues.

The androgen produced in the highest concentration in the body is testosterone (T). This is a 19-C steroid that has androgenic and anabolic effects within the body. T is primarily produced in the gonads but a small amount is produced in the adrenal cortex or from the peripheral conversion of androstenedione. T production is much greater in males than in females (5,000–7,000 μmg/day *versus* 300 μmg/day) [2]. In males, T is primarily produced by the Leydig cells in the testes whereas in females, the primary production of T occurs in the Theca cells of the ovaries. In both sexes, small amounts come from the adrenal cortex and the peripheral conversion of androstenedione.

T acts in the body by acting directly through the AR or indirectly via metabolism to other sex steroids. T can be aromatized to estradiol (E_2_) which activates ER-α and/or ER-β. Alternatively, T can be irreversibly converted to the more potent 5α-dihydrostestosterone (5α-DHT) by the enzyme 5α-reductase. T has many physiological actions in the body. It acts on muscles to stimulate growth and maintenance, it promotes bone development while inhibiting bone resorption, it increases red blood cell and hemoglobin levels, augments libido and erectile function, enhances mood and cognition, and induces lipolysis. Low testosterone levels or deficit in androgen action induces frailty, sarcopenia, poor muscle quality, muscle weakness, hypertrophy of adipose tissue and impaired neurotransmission.

## 3. Myotrophic Action of Androgens

The myotrophic effects of androgens on muscle strength and mass are the main reason for their popularity among androgen users. Androgens also increase lean body mass, decrease fat mass, enhance performance, sustain intensive training periods, and can improve appearance [3,4]. The effects on lean body mass were shown by treating young men with a gonadotropin-releasing hormone (GnRH) analogue that suppressed endogenous T production. These men showed decreased rates of whole body protein synthesis, muscle strength and fat oxidation, together with an increased fat mass [5]. When T was replaced through supplementation, there was a restoration of muscle size and strength with a concomitant reduction in fat mass [6].

Androgens including T increase muscle fiber hypertrophy in human skeletal muscle by enhancing protein synthesis. This occurs via the activation of satellite cells and the promotion of myonuclear accretion when existing myonuclei become unable to sustain further enhancement of protein synthesis [7]. The use of androgen therapy during aging is primarily to promote muscle strength by improving or maintaining muscle mass.

Androgens are used clinically to treat a range of different human disorders. Among these are several catabolic conditions such as obstructive pulmonary disease [8], severe burn injuries [9], and also HIV-related muscle wasting [10]. It can also be used to treat a number of conditions resulting from deficiencies in androgen production, such as constitutional growth retardation and hypogonadism [11]. Androgen therapy can be administered orally, by intramuscular injection, and as gels and creams. Synthetic alkyl esters of androgens have been used therapeutically for decades due to their high potency and prolonged action [12]. The realization that androgen administration can augment muscle hypertrophy has led to the abuse of androgens to increase muscle size, strength and sport performance. One of the earliest accounts of androgen abuse dates back to the 1950s where Soviet weightlifters were allegedly taking testosterone [13]. While the abuse of androgens is more commonly associated with the weightlifting industry, their use is widespread amongst many sports, both at the elite and amateur levels. Alarmingly, androgen use is not limited to adults, with reports that school-aged children use androgens [14].

For bodybuilding and enhanced athletic performance enhancement, it is common that very high doses of androgens are consumed [15]. Moreover, it is common practice to employ “stacking” regimes where a number of different androgens and/or metabolism inhibitors are simultaneously consumed. This can lead to serious clinical consequences such as abnormal liver function, gynocomastia, severe psychological or psychiatric disorders, increased risk of cardiovascular disease; and in females, menstrual disorders and virilization [16]. Many of the androgens that are used are 17-methylated compounds and are associated with high-liver toxicity [17].

## 4. Androgens and the Nutritional Supplement Market

During recent years, the nutritional supplement market has expanded and in 2006, the world-wide market was estimated to be worth more than US$ 60 billion [18]. The use of supplements is popular in the general population; however, athletes corner the market with 44–100% prevalence, dependent on age, gender, level of competition and the type of sport [19]. Whilst vitamins, minerals, proteins and creatine, are not prohibited by WADA, the problem arises when supplements contain additives that are not on their label. This can occur inadvertently through cross-contamination from a production line and/or transport in unclean containers [20] or it can occur through direct addition. This level of contamination can be sufficient to create a positive doping test. For example, 634 nutritional supplements purchased from 13 different countries were analyzed with mass spectrometric methods (GC/MS, LC/MS/MS) for undeclared doping substances and 15% of these minerals, vitamins, creatine or protein supplements contained androgens (mostly prohormones) that were not declared on the label [21]. In another example, bioassays were used to screen a range of nutritional supplements for hormonal activity and found that for 63 supplements, only 13 were negative for estrogenic activity and that only 18 were negative for androgenic activity. All of the other supplements showed agonistic, partial agonistic and/or antagonistic hormone activities, indicating that these health food products contain hormonally active constituents [22]. Since 2002, nutritional supplements have been found to be intentionally spiked with androgens at concentrations higher than 1 mg/g. The androgens found include metandienone [23], stanozolol [24], boldenone [24], oxandrolone and dehydrochloromethyltestosterone [20]. These androgens were either not declared, or were declared but with non-approved names on the labels. The nutritional supplements can be purchased without restriction by telephone order or internet purchase and are delivered by ordinary mail. This allows easy access by the general population for non-medical reasons. Not only do sport nutritional supplements face the problem of androgen spiking, either deliberate or accidental, but “natural” products have also been found to contain hormonally active substances. For example, a herbal product used for the treatment of high level prostate-specific antigen (PSA) was found to contain an estrogenic substance, diethylstilbestrol (DES) using a yeast estrogen bioassay, together with nuclear magnetic resonance (NMR) and liquid chromatography/ time-of-flight MS (LC/TOFMS). This was identifiable clinically because of the development of gynocomastia [25]. Therefore, it is evident that the intentional or unintentional addition of hormonally active substances is an ongoing problem. It is important then that methods are available for the exquisite detection of these hormonally active substances, especially those that are androgenic.

## 5. The Androgen Receptor (AR)

**Figure 1.**
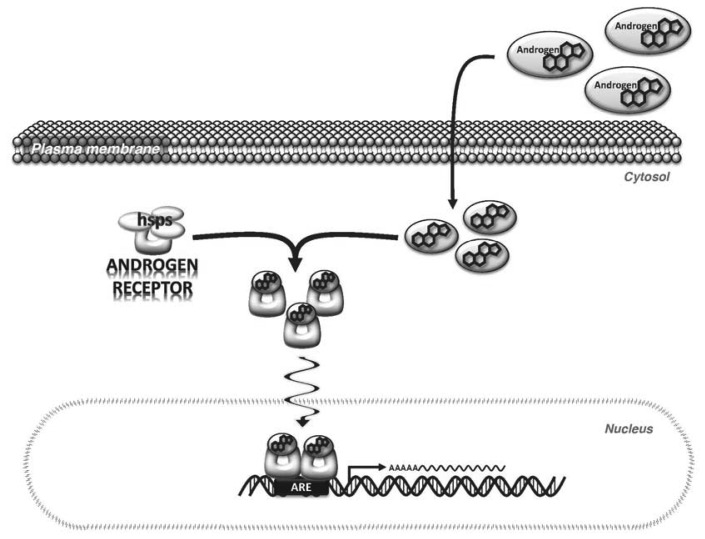
The androgen response in cells. Androgens diffuse through the plasma membrane into the cytosol where they bind to the androgen receptor (AR). The androgen receptor is held in the cytosol by heat shock proteins (HSP) and other cofactors. The binding of androgens to the androgen receptor induces a conformational change in the receptor that frees it from the inhibitory factors and exposes a nuclear localization signal. The androgen/AR complex translocates to the nucleus where the receptor dimerizes and binds to the androgen response elements (ARE) located in the regulatory regions of target genes. Once bound to the DNA, AR enhances gene transcription by RNA polymerase.

Androgens primarily exert their effects by binding to and activating a specific receptor, AR, which is expressed in most cells. The AR is a 110 kDa protein to which the natural androgens, T and DHT, bind with high affinity [26]. In its inactivated state, AR is bound in the cytoplasm to heat shock proteins. When androgens bind to the AR via the ligand binding domain (LBD) of the AR, a conformational change is induced that promotes the dissociation of the heat shock proteins and AR subunits dimerize to form a homodimer. The dimerized AR complex translocates to the nucleus where it binds to androgen response elements (ARE) in the regulatory regions of androgen target genes [27] (Figure 1). Binding of AR to the ARE occurs via the DNA binding domain (DBD). Regulatory cofactors interact with AR to promote DNA binding. Transcriptional activation by AR also involves cofactors that modify the chromatin structure and histone complexes in the DNA surrounding the ARE [26,28]. This is important because the ARE can be located distantly from the transcription start site of androgen-regulated genes, therefore, cofactors that alter DNA shape and flexibility are needed for AR to augment transcription [29].

## 6. Androgen Detection Methods

### 6.1. Mass Spectrometry (MS)

In an effort to control androgen use for enhanced sport performance, WADA screens biological samples for the presence of androgens, metabolites, and/or masking agents. Professional athletes are tested both during, and before, competition. There are a number of routine screening tests that are used to detect exogenous androgen administration, including measuring the testosterone to epitestosterone (T/E) ratio by GC-MS [30]. Epitestosterone is a co-secreted product of T and normally is present in urine at levels similar to testosterone. If exogenous T is administered this will elevate the T level, but not the epitestosterone level. This test is complicated by the fact that the values of testosterone and epitestosterone vary greatly between individuals, and therefore, any sample that meets any one of the following criteria will be sent off for further analysis using Isotope Ratio Mass Spectrometry (IRMS): (1) T/E value greater than or equal to 4; (2) concentration of T or E greater than 200 ng/mL; (3) concentration of androsterone or etiocholanolone greater than 10,000 ng/mL; and (4) concentration of DHEA greater than 100 ng/mL. If a sample is submitted for further evaluation by IRMS, the ^13^C/^12^C ratio of the androgen will be measured. This is because commercially produced androgens will have higher ^13^C levels compared with endogenous androgens [30].

Synthetic androgen use is screened by GC-MS. This is possible because each synthetic androgen has a distinctive chemical structure on GC-MS that is readily identifiable and can be matched to a catalogue kept by WADA. Even trace amounts of synthetic androgen intake are detectable months after the last administration with GC/MS able to detect concentrations in the pg/ml range. The GC-MS screening tests are very sensitive and specific for known androgens on the WADA list. However, these screening tests cannot provide a complete detection of all androgens because they are unable to detect designer androgens.

### 6.2. Receptor Binding (Competitive) Assays

Receptor binding assays are based on the binding affinity of a ligand for its receptor. For this assay, purified receptor is immobilized on a column or suspended in a homogenate and to this, radiolabeled testosterone of known concentration is added. For the test, the suspect molecule is added to the radiolabeled testosterone, and it is measured whether the unknown molecule displaces the binding of the testosterone. If there is displacement then the unknown molecule has AR binding affinity. This type of assay only measures binding to AR and therefore is not able to differentiate between agonist and antagonist activity, or if any activity *per se*. Receptor binding assays require the use of radiolabelling, which presents a hazard if used as a routine screening test. These assays can be developed as high-throughput and are relatively easy to perform. To date, they have been used in a number of applications, including screening animal feed for growth hormones [31] and screening for potential endocrine disrupting chemicals (EDCs) [32,33]. They can also be used to investigate the potential potency of anabolic steroids by assessing the ability to bind to AR [34,35].

### 6.3. Limitation of Structure-Based Methods for Screening of Androgens

It has been discovered that designer androgens have been synthesized with the specific intent of being undetectable by standard screening, allowing athletes to use androgens for enhanced sport performance. For example, in 2002, Catlin *et al.* discovered that norbolethone, a never-before marketed androgen, was present in an athlete's urine sample [36]. It was identified with GC-MS based on chemical signatures of norbolethone that were established in the 1960s when norbolethone was tested in clinical trials but later not marketed. Since then, other designer androgens have been detected, primarily found in nutritional supplements, marketed illicitly over the internet. In 2003, tetrahydrogestrinone (THG), a modified progestin, was discovered. A yeast-based *in vitro* androgen bioassay confirmed that THG was a potent androgen, capable of activating AR [37]. Later, THG was detected in a human urine sample using a bioassay in combination with LC/TOFMS [38]. Structural modification of either the T or progesterone background could produce an almost limitless number of androgenic steroids with structures that are not on the WADA detection list [39]. Also in development for clinical use are non-steroidal androgenic molecules (SARMs) that too will pose a risk to sports doping because of their ability to activate the AR.

## 7. Androgen Bioassays

Androgen bioassays used for detection differ from the techniques described above because they mimic AR function and are not dependent on chemical structure. There are a number of different bioassays ranging from those based on whole animals to those based on mammalian or yeast cells. The Hershberger assay is an example of an *in vivo* androgen bioassay. The endpoint of this assay is a measured increase in the weight of androgen-dependent tissues. It is based on orchidectomised animals that produce little endogenous sex steroid hormones. These animals are treated with the test compound. If the test compound is androgenic, it will promote growth of androgen-dependent tissues [40]. As this is an *in vivo* assay, metabolism of test molecules can also be tested by analyzing metabolites present in the blood stream and/or urine. As metabolism occurs upon treatment, this assay cannot be used to screen for activation or inactivation of androgens but it does allow the dissection of anabolic and androgenic effects of the test molecule or its metabolites. An assay based in animals is not feasible for routine sports doping screening in WADA laboratories. This has led to the development of *in vitro* cell-based androgen bioassays to screen for androgenic compounds.

*In vitro* cell-based bioassays are widely used to detect androgenic molecules. They were first developed to test environmental pollutants (endocrine disrupting chemicals, EDCs) for their ability to alter normal hormonal function. Many substances including detergents (nonylphenol and other alkylphenols), plastics (bisphenol A), pesticides, insecticides, and even pharmaceutical wastes such as birth control tablets (ethinylestradiol) are now classified as EDCs [41]. In vitro yeast androgen bioassays can be used in combination with other detection methods such as ultra high performance liquid chromatography combined with time-of-flight-tandem mass spectrometry (UHPLC/TOFMS) or liquid chromatography screening method. In the first example, herbal mixtures and sport supplements were screened using the yeast yEGFP bioassay, after sample preparation to activate inactive pro-androgens, androgen esters and conjugated androgens. The samples that tested positive were then analysed by UHPLC/TOFMS which led to the positive identification of nortestosterone phenylpropionate, testosterone cyclohexanecarboxylate and methyltestosterone in herbal supplements and methylboldenone, testosterone and the androgen esters, methyltestosterone propionate or testosterone isobutyrate, testosterone buciclate, and methylenetestosterone acetate. In the second example, 18 different dietary supplements that had been previously analysed with liquid chromatography-tandem mass spectrometry method (LC-MS/MS) were screened with the yeast yEGFP bioassay. LC-MS/MS showed 11 samples positive for androgens- a finding that was corroborated by the bioassay. However, of the seven that were negative using LC-MS/MS, two were positive when screened by the bioassay. Subsequently, LC/TOFMS identified 4-androstene-3β,17β-diol and 5α-androstane-3β,17β-diol. These assays show that bioassay guided analysis is a useful procedure to detect, isolate, and identify unknown androgens in suspected samples. Moreover, it shows that bioassays can detect androgens in samples where LC-MS/MS could not, highlighting that bioassays have a valuable role in the fight against doping.

*In vitro* cell-based bioassays are also used to test for illegal substances such as growth promoters that are given to livestock. These androgens or androgen-like molecules enhance the growth of the animals and are used as a means to increase profit. Naturally occurring steroids such as testosterone or synthetic androgens such as 19-nortestosterone, trenbolone acetate, and medroxyprogesterone are used to illicitly augment growth of animal livestock [42,43]. In addition, prohormones such as dehydroepiandrosterone (DHEA) are being used, and these are often hard to detect if they have been exogenously administered [44]. GC-MS-based screening of material such as meat extracts, feed or urine may fail to detect these substances because deconjugating steps are required prior to GC-MS analysis and these processing steps can destroy the structures. GC-MS may also fail to detect novel structures or those with unknown metabolic profiles. Bioassays can complement the screening of samples as they are capable of detecting hormonally active compounds in prepared extracts and if the appropriate host cell is used for the bioassay such as hepatocytes then such assays may also detect prohormones [43,45,46]. For these reasons, bioassays are ideal for the detection of androgens or proandrogens added to nutritional supplements.

### Cell-Based Androgen Bioassays

#### Cell Proliferation Assays

Cell proliferation assays can be used to measure the hormonal activity of a suspected agonist (or antagonist) in a sample because hormones, via their specific receptors, stimulate cell growth. In these assays, radioactive-labeled nucleotides are included in the culture media that become incorporated into DNA as cells proliferate. The radiolabel that incorporates into cells is a direct measurement of cell proliferation. These assays can measure both agonist and antagonist activity, as an agonist for the receptor of interest can stimulate cell growth, whereas an antagonist will block cell growth in the presence of an agonist. To date, this type of bioassay has not been extensively used to screen for androgens, however, it is the basis of the E-screen, which uses the human breast-cancer cell line (MCF-7) to screen for xenoestrogens [47]. This assay is relatively simple to perform and is amenable to high-throughput readouts. However, results can be confounded by cell expression of other receptors (such as AR and glucocorticoid receptor) that induce non-specific cell proliferation. The assay relies on cell growth and, therefore, it can take days to produce results [48]. Thus, it is not really feasible for use in sport doping laboratories.

#### *In Vitro* Androgen Receptor-Reporter Gene Bioassays

**Figure 2.**
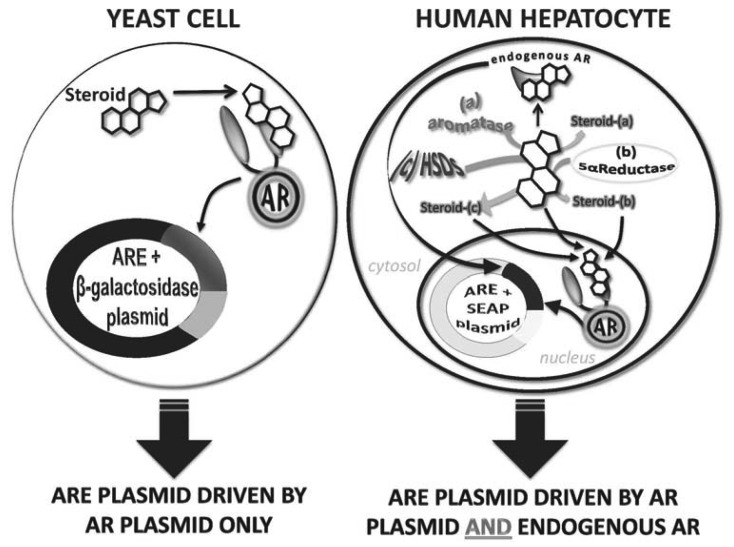
Yeast and mammalian cell-based androgen bioassays. Both bioassays are based on the transfection of two plasmid vectors. One plasmid vector is an androgen receptor (AR) expression vector that allows high level constitutive expression of AR within cells that contain no (yeast) or low level (mammalian) of endogenous AR. The second plasmid vector is an androgen response element (ARE) driven reporter gene vector. For yeast cells, the most efficient reporter gene, to date, is β-galactosidase. For mammalian cells, a number of reporter genes are used, with secreted alkaline phosphatase (SEAP) a favored choice because of its ease of detection. Yeast cells do not express androgen metabolizing enzymes. Mammalian cells such as human hepatocytes express a number of different metabolizing enzymes including aromatase, 5α-reductase and hydroxysteroid reductases (HSD).

*In vitro* androgen receptor-reporter gene bioassays exploit the natural signaling pathway of androgens. Firstly, a host cell is chosen because it minimally or does not express endogenous AR. The host cell is then genetically manipulated to express AR. Secondly, the host cell is transformed with a reporter vector in which a minimal promoter is driven by either AREs or MMTV elements (that also bind AR) that, together, regulate the expression of a reporter enzyme or protein (e.g., luciferase, green fluorescent protein (GFP), secreted alkaline phosphatase (SEAP) or β-galactosidase). When the genetically transformed cell is exposed to androgens, the androgens activate AR that, in turn, binds to the cloned ARE or MMTV and drives the expression of the reporter enzyme or protein, which can easily be assayed (Figure 2).

The two major host cells are yeast or mammalian cells. The advantages of the yeast cell-based bioassay are that yeast cells are relatively easy and cheap to grow and are suitable for high-throughput applications. To date, yeast cell bioassays have been used to test urine samples for androgen detection, nutraceutical extracts for androgen detection, cattle hair samples for steroid esters as well as testing for the androgenic potency of pure preparations of many androgens, designer androgens and progestins [37,49–52]. The most commonly used yeast cell-based bioassay is that in which the laboratory yeast strain, *Saccharomyces cerevisiae*, is co-transformed with an AR expression vector and an ARE/β-galactosidase reporter gene vector [53]. For this assay, the yeast cells are cultured in selective minimal media and then exposed to the steroid/steroid extract of interest for 24 h before cells are lysed and β-galactosidase activity measured. This approach is reliable and reproducible, with an EC_50_ of ∼5 nM so therefore able to measure T concentrations within a male physiological range, although they are less sensitive than GC-MS that is able to detect steroids in the pM range. Yeast cells are exceptional for monitoring pure androgenic potential of test molecules or extracts as they do not express metabolizing enzymes. This, however, means that yeast cell based assays will not detect activity of any molecule that requires activation (that is a prohormone) or a molecule that may be inactivated *in vivo*. Therefore, yeast cell-based assays *per se* cannot be used as a sole means of androgen detection of unknown samples.

The traditional AR/ARE/β-galactosidase reporter assay involves long incubation times (24 h), pre-assay preparation and post-incubation cell lysis steps. To try and improve on this assay, other reporter genes have been used in yeast cell androgen bioassays. For example, luciferase has been used as its detection requires no cell lysis step with the substrate, luciferin, directly added to the yeast culture for the measurement of enzyme activity [54]. This assay is quick, easy to use, however, it has lower sensitivity with an EC_50_ of ∼10 nM for T [54]. Both the luciferase and β-galactosidase enzyme-based assays may have issues due to the build-up of artifacts, or if the compounds tested inhibit enzyme activity or stabilize the enzyme [55]. To address these issues, a simple fluorescent measurement to screen has been generated that utilizes an ARE-yEGFP (yeast enhanced green fluorescent protein) reporter construct. yEGFP as the reporter is superior to β-galactosidasae or luciferase because no enzyme substrate is required instead just a simple fluorescent readout, making the assay cheaper, quicker and easier to complete. However, this assay shows a lower sensitivity with an EC_50_ of 50 nM for T [45,51]. Therefore, to date, it remains that the most sensitive of the yeast cell-based bioassays is that based on the original AR/β-galactosidase enzyme in *S. cerevisiae.*

#### *In Vitro* Mammalian Cell-Based Androgen Bioassays

In similar fashion to yeast cell-based bioassays, a number of mammalian cell bioassays have been developed. These harbor different reporter enzymes/proteins and/or are established in different cell types. The mammalian cell-based bioassays involve stable transfection of the cell with an AR expression vector and an ARE/minimal promoter/reporter gene vector. The reporter enzyme/protein used for mammalian cell assays include luciferase, green fluorescent protein and SEAP (see Table 1). A common feature of mammalian cell-based bioassays is that they show higher sensitivity than yeast cell-based bioassays, with reported EC_50_ values often 10-fold lower. However, due to the expression of endogenous receptors they show lower specificity because of crosstalk between receptors [56]. Moreover, mammalian cells express a range of androgen metabolizing enzymes including aromatase, 5α-reductase, 17β-hydroxysteroid reductase and 3α-hydroxysteroid reductase [52], that can alter the potency of the test substrate, either activating it making it a stronger androgen or deactivating it making it a weak androgen. The benefits of the metabolizing enzymes are that they allow for prohormones to be detected as well as offering insight into how a test steroid/extract may behave *in vivo*. However, the metabolism capacity of mammalian cells may be limited by the type of host cell as different cell types express metabolizing enzymes at different levels and the passage number of the cell culture as some cells are reported to switch off expression of some metabolizing enzymes during *in vitro* culture. Thus, *in vitro* metabolism in cultured cells may not reflect *in vivo* metabolism. To date, the literature reports ambiguity with EC_50_ sensitivities and specificities most likely due to these metabolizing effects [57–63]. Testosterone, for example, has been shown to exhibit very sensitive responses using a mouse mammary tumor virus (MMTV)/luciferase construct in human kidney cells (HEK293) with an EC_50_ of 0.3 nM [57]. By contrast, this same construct is less sensitive in Chinese hamster ovary (CHO) cells with an EC_50_ of 1.06 nM [58]. In a recent study by Akram *et al*. [52], the importance of choosing the right reporter construct can be further emphasized, whereby even using the same cell type (HEK293), the EC_50_ values of the tested androgens differed between the MMTV/luciferase bioassay and the enhancer/ARE/SEAP showing EC_50_s of 136 ± 60 nM and 450 ± 24 nM respectively for a known androgen, formadrol. Reported variations in sensitivities across bioassay types (yeast and mammalian) for various androgens are shown in Table 1. Thus, mammalian cell-based assays are more sensitive than yeast cell-based assays, however due to the potential metabolism of androgens they can be less robust than yeast cells in defining a true androgenic potency and results may be confounded by metabolism effects that must be considered [52] and for a detailed analysis of a test extract/steroid are best combined with a yeast cell-based assay to determine baseline androgen potency.

The detection of prohormones or an understanding of potential metabolism of test extracts is central for the analysis of nutritional sport supplements. Mammalian cell lines may be limited in their metabolic capacity, compared to *in vivo* metabolism. To address this, a liver tissue metabolism step has been combined with the yeast AR/ARE/EGFP-based bioassay [64,65]. In this two-step assay, test extracts are first incubated with a bovine liver S9 fraction, the extract recovered, and then exposed to the yeast AR bioassay. As the liver tissue is whole, it is expected that this *ex vivo* approach will mimic the *in vivo* capacity for enzymatic conversions of steroids and therefore detect both prohormones and/or strong androgenic metabolites. In another example of introducing a pre-metabolism step prior to testing with a yeast AR bioassay to allow for prohormone or androgen metabolite detection, samples were pre-treated with a *Helix pomatia* enzyme mix to activate inactive hormone conjugates including sulphates, glucuronides and glycosides [46]. This example was in the setting of feed supplementation, rather than nutraceutical supplements, however, it is possible that a similar approach could be used to detect such conjugates if they were components of nutraceuticals.

## 8. Feasibility Issues with Current Cell Based Androgen Bioassays

At present no androgen bioassay has been endorsed by sport doping laboratories for high throughput screening for androgens in nutritional sports supplements or biological samples. Yeast cell-based bioassays are exceptional because of their specificity, ease of use (especially the yEGFP-based system), and they are cost-effective. The downside of yeast cell-based bioassays are they are less sensitive than mammalian cell-based bioassays, and they are unable to detect prohormones or potent androgenic metabolites because they have no metabolic capacity. To this end, the combination of using mammalian cell-based bioassays or liver tissue metabolism steps with the yeast cell-based bioassay may be the best approach for detection of androgens, potential androgenic metabolites, and proandrogens in nutritional sports supplements. Mammalian cells may be limited in their capacity to mimic *in vivo* metabolism with differential (and perhaps decreasing) expression of necessary metabolizing enzymes. Liver tissue may be less limited in the full complement of metabolizing enzymes, however, this approach requires animal sacrifice to obtain liver slices and this may therefore limit the feasibility of this approach for high throughput screening in WADA-accredited laboratories.

## 9. Conclusions

Current WADA screening methods, such as the highly sensitive GC-MS, fail to detect designer androgens of unknown structure. Novel designer androgens are being increasingly incorporated in nutritional supplements, allowing athletes to use androgens to enhance performance. *In vitro* androgen bioassays are able to detect designer androgens, and in some cases, proandrogens, as they do not depend on structure analysis, but instead exploit the natural pathway of androgen signaling via AR activation. While not as sensitive as GC-MS, some *in vitro* androgen bioassays can detect physiological concentrations of testosterone, and therefore, can detect androgens at the supraphysiological levels used in sports doping. Importantly, a combination of yeast and mammalian cell-based or liver tissue and yeast AR bioassays can comprehensively screen for known androgens, designer androgens, and proandrogens. Therefore, further development of *in vitro* androgen bioassays to decrease testing times, processing steps such as further development of yEGFP assays and improved detection limits (sensitivity) could help to combat the war against androgen use. By incorporating routine bioassay screening of nutritional supplements, blatant and inadvertent androgen doping may be reduced.

## Figures and Tables

**Table 1.** Sensitivities of various androgen bioassays.

**Androgen**	**Construct**	**Cell line/species**	**EC_50_(nM)**	**Author**
Testosterone	MMTV/Luciferase AR	HEK293	0.3	Roy, P. 2006 [56]
	ARE/Luciferase	U2-OS	0.86	Houtman, C. 2009 [57]
	ARE/Luciferase	CHO	1.06	Araki, N. 2005 [58]
	ARE/β-galactosidase	*S. cerevisiae*	5	Death, A. 2005 [52]
	ARh-LBD-ASC1/ β-galactosidase	*S. cerevisiae*	15	Lee, H. 2003 [59]
	ARE/GFP	*S. cerevisiae*	23	Beck, V. 2008 [60]
				
5α-Dihydrotestosterone (DHT)	ARE/Luciferase	CHO	0.22	Araki, N. 2005 [58]
	ARh-LBD-ASC1/ β-galactosidase	*S. cerevisiae*	4.8	Lee, H. 2003 [59]
	ARE/GFP	*S. cerevisiae*	16	Beck, V. 2008 [60]
				
Methyltestosterone	ARE/Luciferase	CHO	0.7	Araki, N. 2005 [58]
	ARE/GFP	*S. cerevisiae*	1.2	Beck, V. 2008 [60]
				
4-Androstenedione	ARE/Luciferase	CHO	1.02	Araki, N. 2005 [58]
	AR-CALUX	U2-OS	4.5	Sonneveld, E. 2005 [61]
	ARE/Luciferase	*S. cerevisiae*	500	Michelini, E. 2005 [62]
